# Accuracy of Zika virus disease case definition during simultaneous Dengue and Chikungunya epidemics

**DOI:** 10.1371/journal.pone.0179725

**Published:** 2017-06-26

**Authors:** José Ueleres Braga, Clarisse Bressan, Ana Paula Razal Dalvi, Guilherme Amaral Calvet, Regina Paiva Daumas, Nadia Rodrigues, Mayumi Wakimoto, Rita Maria Ribeiro Nogueira, Karin Nielsen-Saines, Carlos Brito, Ana Maria Bispo de Filippis, Patrícia Brasil

**Affiliations:** 1 Escola Nacional de Saude Publica, Fundacao Oswaldo Cruz, Rio de Janeiro, Brazil; 2 Instituto de Medicina Social, Universidade do Estado do Rio de Janeiro, Rio de Janeiro, Brazil; 3 Instituto Nacional de Infectologia Evandro Chagas, Fundacao Oswaldo Cruz, Rio de Janeiro, Brazil; 4 Laboratorio de Referencia de Flavivirus, Instituto Oswaldo Cruz, Fundacao Oswaldo Cruz, Rio de Janeiro, Brazil; 5 David Geffen UCLA School of Medicine, Los Angeles, United States of America; 6 Departmento de Clinica Medica, Universidade Federal de Pernambuco, Recife, Brazil; Singapore Immunology Network, SINGAPORE

## Abstract

**Background:**

Zika is a new disease in the American continent and its surveillance is of utmost importance, especially because of its ability to cause neurological manifestations as *Guillain-Barré* syndrome and serious congenital malformations through vertical transmission. The detection of suspected cases by the surveillance system depends on the case definition adopted. As the laboratory diagnosis of Zika infection still relies on the use of expensive and complex molecular techniques with low sensitivity due to a narrow window of detection, most suspected cases are not confirmed by laboratory tests, mainly reserved for pregnant women and newborns. In this context, an accurate definition of a suspected Zika case is crucial in order for the surveillance system to gauge the magnitude of an epidemic.

**Methodology:**

We evaluated the accuracy of various Zika case definitions in a scenario where Dengue and Chikungunya viruses co-circulate. Signs and symptoms that best discriminated PCR confirmed Zika from other laboratory confirmed febrile or exanthematic diseases were identified to propose and test predictive models for Zika infection based on these clinical features.

**Results and discussion:**

Our derived score prediction model had the best performance because it demonstrated the highest sensitivity and specificity, 86·6% and 78·3%, respectively. This Zika case definition also had the highest values for auROC (0·903) and R^2^ (0·417), and the lowest Brier score 0·096.

**Conclusions:**

In areas where multiple arboviruses circulate, the presence of rash with pruritus or conjunctival hyperemia, without any other general clinical manifestations such as fever, petechia or anorexia is the best Zika case definition.

## Introduction

To the present date 75 countries and territories have reported evidence of mosquito-borne ZIKV transmission since 2007 [1]. ZIKV continues to disperse, reinforcing the need for surveillance in countries with vector circulation. Twenty-nine countries or territories have reported microcephaly and other CNS malformations suggestive of congenital Zika infection. In addition, an increased incidence of GBS was reported by 21 countries and territories. In non-endemic countries imported cases and sexual transmission of ZIKV were also identified by health authorities [1].

Zika infection has affected countries endemic for other arboviruses, particularly Dengue and Chikungunya. The oldest and most frequent of arboviruses, Dengue has caused epidemic outbreaks for decades, with WHO estimates of more than 100 million cases per year in 100 countries where the disease is endemic [2]. Dengue is associated with risks of severe disease and deaths, but is not frequently associated with neurological complications or fetal malformations. In Brazil in 2015 there were 1,500,578 cases, with 833 deaths [3].

In 2005/2006, large outbreaks of Chikungunya took place in many countries where Dengue is endemic, starting in the Indian Ocean and Indian Islands, and by 2013 reaching the Western Hemisphere of the Caribbean island of Saint Martin. By July 2014 Chikungunya had spread to another 20 countries in the Caribbean and Central and South America, with more than 500,000 cases reported in 2016 [4, 5]. Chikungunya has as main complications significant disease severity and deaths occurring in the acute phase of illness, with frequent neurological complications [6]. Unlike the other two arboviruses, Chikungunya often results in chronic forms of disease, with about 40% of acute phase patients progressing to chronic arthritis for months to years [7].

PAHO/WHO warned of the risk of simultaneous circulation and potential outbreaks with more than one arbovirus, with epidemiological surveillance being the key element for identification and monitoring of clinically compatible cases for each one of the three arboviruses [8].

The major challenge for an epidemiological surveillance system where the three viruses are circulating simultaneously is the initial case detection in an epidemic based on the identification of a clinical picture compatible with the definition of a suspected case. With the progression of concurrent epidemics due to different co-circulating arboviruses, the surveillance system should be able to differentiate between distinct illnesses in order to enable adequate case reporting so that the data reflects the real epidemiological situation. This will allow for adequate prediction of potential complications due to each arbovirus.

The laboratory confirmation of the diagnosis of ZIKV infection can be difficult especially in countries endemic for other arboviruses such as Brazil. Detection of the virus by reverse transcription polymerase chain reaction (RT–PCR) is not always available and reference laboratories may be overloaded during epidemics. Besides, virus load levels may be low with sensitivity of molecular tests also being low [9, 10], also due to a relatively short window of detection of virus in blood or urine. ZIKV cross-reacts immunologically with Dengue and other flaviviruses, wich frequently co-circulate, thus complicating the interpretation of serological results.

Zika virus infection is the most recent of the three epidemics, and its clinical pattern of disease has been newly described. Different case definitions have been published, often based on small case series. Consequently the sensitivity of some case definitions has been questioned by clinicians [11]. For the diagnosis of Zika to be made using clinical and epidemiological criteria in resource-limited areas, or during epidemic periods, it is necessary to establish a sensitive case definition with good specificity.

An accurate case definition would optimize the allocation of human and financial resources according to the distribution of notifications and permit the prioritization of higher risk groups, such as pregnant women, or the monitoring of complications. Furthermore, it would be useful to the surveillance of imported cases in non-endemic areas and the development of clinical management protocols. Case definitions which are non-specific but with a high sensitivity can overestimate the Zika virus infection burden in a population. In this scenario, the rate of potential complications of ZIKV infection such as GBS or congenital Zika virus syndrome will be underestimated, as many of the reported "cases" would not have been true Zika cases in the first place. For example, if 10,000 cases of ZIKV are reported because of an overly sensitive case-definition, but the true number of cases is 1000, with an estimated frequency of GBS of 1% and adverse pregnancy outcome frequency of 40%, the true number of GBS cases would be 10 cases and the number of cases with adverse pregnancy outcomes 400. However, because the number of reported cases of Zika is erroneously estimated as 10,000, it appears that the frequency of GBS is 0.1% and that of adverse pregnancy outcomes 4% due to the larger denominator. This in fact has generated large discrepancies across different studies evaluating the frequency of ZIKV-related complications.

Thus, the aim of this study was to discuss the accuracy of existing case definitions of ZIKV infection and propose, based on our experience, a novel and effective case definition to be used particularly in situations where triple epidemics overlap.

## Materials and method

### Study population and eligibility criteria

Patients were recruited from August 2014 to May 2016 in the outpatient clinic for Acute Febrile Diseases (AFD) of the Instituto Nacional de Infectologia Evandro Chagas of the Oswaldo Cruz Foundation. During this period, all patients with a febrile illness or rash without an evident focus of infection who sougth care within seven days of onset of symptoms were invited to participate in the study.

Considering the period of documented local transmission of DENV, ZIKV and CHIKV in the municipality of Rio de Janeiro, we selected for this analysis only patients who had RT-PCR results available for confirmation of diagnosis during the epidemic period. Thus, we included patients evaluated between Aug 1 2014 and Dec 31 2014 if they had RT-PCR results for DENV; patients examined from Jan 1 2015 to Oct 31 2015 with RT-PCR results for DENV and ZIKV; and patients seen between November 1 2015 and July 31 2016 if they had RT-PCR results for DENV, ZIKV and CHIKV. Refusal to participate, absence of recorded physical examination and absence of laboratory test results were exclusion criteria.

### Study design

A cross-sectional study was conducted in our outpatient population to enable the development of a clinical prediction model of ZIKV infection based on the presence of signs and symptoms. Besides the development of the derivation model, this approach allowed us to evaluate the diagnostic performance of the model and compare results with case definitions of suspected Zika proposed for surveillance by different public health organizations.

### Clinical signs and symptoms and laboratory confirmation of infection

Presence of the following clinical features were assessed: maculopapular rash, itching, edema, macular rash, enanthema, lymphadenopathy, arthralgia, conjunctival hyperemia, oropharyngeal pain, earache, nasal congestion, purpura, fever, vomiting, hepatomegaly, abdominal pain, nausea, anorexia, headache, taste alteration, bleeding, prostration, lightheadedness, chills, myalgia, dyspnea, low back pain, cough, coryza diarrhea, gingivorrhagia, sweating, petechiae, hoarseness, choluria, dysuria, photophobia, retro-orbital pain and epistaxis. These signs and symptoms were evaluated in the first medical visit by history and clinical examination recorded in structured case report forms. Data on signs and symptoms present on the first or second clinic visit within the first week of disease was collected.

Confirmatory diagnosis of infection for ZIKV, DENV and CHIKV was made by real time PCR test of blood or urine specimens obtained during the same time period [10]. Dual or triple co-infections were excluded from analysis. Other febrile illnesses (OFI) were diagnosed based on negatives PCR results for all circulating arbovirus. Patients included before 2015 and those who tested negative for ZIKV by RT-PCR were classified as Zika-negative.

### Development of the clinical prediction model and its performance

Odds ratio (OR) was used as the association measure to identify the signs and symptoms related to ZIKV infection. Crude and adjusted estimates in single and multiple regression models were obtained using STATA 13·0 software. 95% confidence intervals (CI) were calculated using the Mantel-Hanszel method. Explanatory variables with a marginal association with the outcome (p≤0·20) in the bivariate analysis were included into the multiple regression models. A stepwise multiple logistic regression was conducted with backward selection to identify the prediction model, with a significance level of 5%.

### Clinical prediction model and evaluation of suspected case definitions

We compared the predictive models that presented the best fit by the likelihood ratio test (LR test) and R^2^ (coefficient of determination). We studied six (6) suspected case definitions of Zika used in epidemiological surveillance by the following groups: the Pan American Health Organization (PAHO) 2015 [12], PAHO 2016 [13], the European Center for Disease Prevention and Control (ECDC) 2016 [14], the Centers for Disease Control and Prevention (CDC) 2016 [15], the World Health Organization (WHO) 2016 [16] and the case definition used by the Brazilian Ministry of Health in 2016 [17]. A score based rule derived from the selected prediction model was evaluated and compared with the Zika suspected case definitions. The scores were defined by the ratio between the odds ratio for each associated signal and the one of smaller magnitude of the derived model. The comparisons were done using measures of accuracy sensitivity, specificity, diagnostic odds ratio, area under receiver operating characteristic (auROC) curve and corresponding 95% confidence intervals. The best cut-off value for the score was determined by the Youden index that maximizes the sum of sensitivity and specificity.

### Ethical issues

This study was approved by the Institutional Review Board of the Instituto Nacional de Infectologia Evandro Chagas of the Oswaldo Cruz Foundation.

## Results

### Socio-demographic characteristics of the study population

Among 659 participating subjects, 138 were positive by RT-PCR for ZIKV, 113 for DENV, 45 for CHIKV and 363 were diagnosed with OFI. The laboratory confirmation of cases was based on RT-PCR tests, which were performed for all circulating arboviruses in each period result of all cases. No patients included in the study had dual or triple infections. The mean age of the study population was 37 years (range: 9 to 78 years for Zika; 36 years (range 10 to 66 years for dengue; 43 years (range 17 to 72 years for chikungunya and 35 years (range 4 to 80 years for OFI).

Among subjects positive for ZIKV, 90 (65·2%) were women, 88 (63·8%) self-reported as white and 46 (33·3%) had a university degree. For dengue and OFI, most cases were in males and the majority of the study population self-reported as white and about one third reported having a university degree (Table 1).

**Table 1:** Socio-demographic characteristics of the population studied according to the presence of Zika, dengue or chikungunya diagnosis.

Characteristics	Zika		Dengue		Chikungunya		OFI	
	N	%	N	%	N	%	N	%
Number of participants	138		113		45		363	
Age (years)								
Median		37·3		35·7		42·7		35·7
Min		9·6		10·7		17·5		4·7
Max		78·7		66·3		72·0		80·2
no data	0		0		7		1	
Gender								
Male	48	34·8	69	61·1	21	46·7	206	56·8
Female	90	65·2	44	38·9	24	53·3	157	43·3
no data	0		0		0		0	
Race								
White	88	63·8	75	66·4	17	37·8	252	69·4
Black	5	3·6	11	9·7	3	6·7	31	8·5
Asian	2	1·5	1	0·9	10	22·2	9	2·5
Mixed race	23	16·7	20	17·7	0	0·0	50	13·8
Other	0	0·0	0	0·0	1	2·2	3	0·8
no data	20	14·5	6	5·3	14	31·1	18	5·0
Education (years of schooling)								
illiterate	0	0·0	2	1·8	1	2·2	4	1·1
< 8	9	6·5	18	15·9	9	20·0	38	10·5
8	6	4·4	11	9·7	2	4·4	27	7·4
9 to 10	19	13·8	12	10·6	2	4·4	27	7·4
11	36	26·1	25	22·1	14	31·1	75	20·7
High School Degree	14	10·1	11	9·7	6	13·3	44	12·1
University Degree	46	33·3	32	28·3	9	20·0	130	35·8
no data	8	5·8	2	1·8	2	4·4	18	5·0

### Clinical signs and symptoms associated with ZIKV disease

The simple logistic regression results are presented in Table 2.

**Table 2:** Clinical signs and symptoms associated with ZIKV infection.

Feature	ZIKV positive		ZIKV negative		OR	
	N	%	N	%		95%CI
Macular rash						
Yes	89	64·5	202	42·2	2·49	1·68–3·68
No	49	35·5	277	57·8	reference group*	
Maculopapular rash						
Yes	65	48·1	28	5·9	14·75	8·86–24·56
No	70	51·9	445	94·1	*reference group*	
Itching						
Yes	110	79·7	144	28·5	9·87	6·24–15·60
No	28	20·3	362	71·5	*reference group*	
Prostration						
Yes	108	78·3	439	86·4	0·56	0·35–0·91
No	30	3·0	69	13·6	*reference group*	
Headache						
Yes	95	68·8	440	85·3	0·38	0·24–0·58
No	43	31·2	76	14·7	*reference group*	
Arthralgia						
Yes	96	69·6	290	56·3	1·77	1·18–2·65
No	42	30·4	225	43·7	*reference group*	
Myalgia						
Yes	86	62·3	404	78·1	0·46	0·30–0·69
No	52	37·7	113	21·9	*reference group*	
Conjunctival hyperemia						
Yes	74	53·6	178	35·9	2·06	1·41–3·02
No	64	46·4	318	64·1	*reference group*	
Low back pain						
Yes	74	53·6	317	63·9	0·65	0·44–0·95
No	64	46·4	179	36·1	*reference group*	
Retro-orbital pain						
Yes	74	53·6	266	51·9	1·07	0·73–1·56
No	64	46·4	247	48·1	*reference group*	
Lymphadenopathy						
Yes	55	39·9	118	23·6	2·15	1·44–3·20
No	83	60·1	383	76·4	*reference group*	
Chills						
Yes	61	44·2	325	64·1	0·44	0·30–0·64
No	77	55·8	182	35·9	*reference group*	
Fever						
Yes	90	69·2	474	95·8	0·09	0·05–0·17
No	40	30·8	21	4·2	*reference group*	
Anorexia						
Yes	56	40·6	359	70·3	0·29	0·19–0·42
No	82	59·4	152	29·7	*reference group*	
Photophobia						
Yes	56	40·6	218	43·8	0·87	0·59–1·28
No	82	59·4	280	56·2	*reference group*	
Oropharyngeal pain						
Yes	45	32·6	119	23·5	1·57	1·04–2·37
No	93	67·4	387	76·5	*reference group*	
Edema						
Yes	31	22·6	39	7·7	3·49	2·08–5·85
No	106	77·4	466	92·3	*reference group*	
Taste alteration						
Yes	42	40·8	237	64·1	0·39	0·25–0·60
No	61	59·2	133	36·0	*reference group*	
Nausea						
Yes	36	26·1	287	56·7	0·26	0·17–0·40
No	102	73·9	219	43·4	*reference group*	
Enanthema						
Yes	16	11·7	27	5·6	2·24	1·17–4·29
No	121	88·3	458	94·4	*reference group*	
Bleeding						
Yes	5	3·6	64	12·4	0·26	0·10–0·67
No	133	96·4	452	87·6	*reference group*	
Petechiae						
Yes	10	7·2	59	11·8	0·58	0·29–1·17
No	128	92·8	443	88·2	*reference group*	
Purpura						
Yes	0	0·0	7	1·4	—	
No	138	100·0	494	98·6	*reference group*	
Gingivorrhagia						
Yes	2	1·4	22	4·4	0·32	0·07–1·38
No	136	98·6	480	95·6	*reference group*	
Epistaxis						
Yes	2	1·4	12	2·3	0·62	0·14–2·78
No	136	98·6	503	90·7	*reference group*	
Nasal congestion						
Yes	27	20·0	79	16·0	1·32	0·81–2·14
No	108	80·0	416	84·0	*reference group*	
Sweating						
Yes	30	22·1	136	46·3	0·32	0·20–0·52
No	106	77·9	158	53·7	*reference group*	
Diarrhea						
Yes	34	24·6	165	32·4	0·68	0·44–1·04
No	104	75·4	344	67·6	*reference group*	
Abdominal pain						
Yes	31	22·8	235	54·5	0·24	0·15–0·38
No	105	77·2	196	45·5	*reference group*	
Cough						
Yes	24	17·4	134	26·2	0·59	0·36–0·96
No	114	82·6	378	73·8	*reference group*	
Coryza						
Yes	24	17·4	137	27·1	0·56	0·35–0·91
No	114	82·6	369	72·9	*reference group*	
Lightheadedness/ fainting						
Yes	26	19·0	190	37·3	0·39	0·24–0·62
No	111	81·0	319	62·7	*reference group*	
Hoarseness						
Yes	9	6·5	46	9·3	0·68	0·32–1·43
No	129	91·5	451	90·7	*reference group*	
Earache						
Yes	12	8·7	38	7·7	1·13	0·57–2·23
No	126	91·3	453	92·3	*reference group*	
Dysuria						
Yes	8	5·8	36	8·7	0·64	0·29–1·42
No	130	94·2	377	91·3	*reference group*	
Choluria						
Yes	24	17·4	99	19·7	0·85	0·52–1·40
No	114	82·6	404	80·3	*reference group*	
Dyspnea						
Yes	9	6·5	63	12·3	0·49	0·24–1·03
No	129	93·5	451	87·7	*reference group*	
Vomiting						
Yes	5	3·6	114	22·2	0·13	0·05–0·33
No	132	96·4	399	77·8	*reference group*	
Hepatomegaly						
Yes	2	1·5	33	6·7	0·20	0·04–0·86
No	135	98·5	458	93·3	*reference group*	

* Reference group = the ausence of the sign or sympton

### Clinical prediction models of ZIKV disease

**Fig 1:**
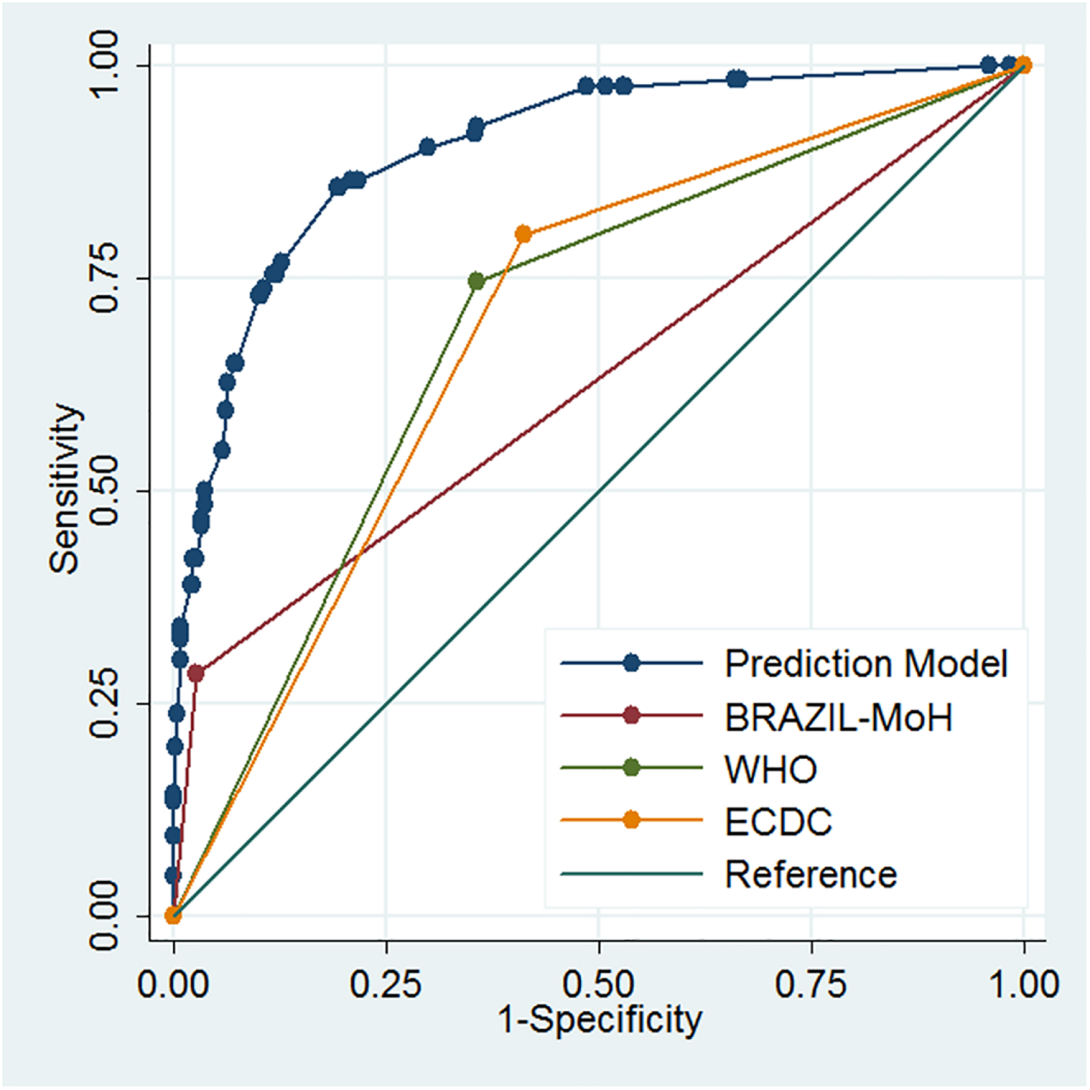
Prediction models area under ROC based on signs and symptoms of ZIKV infection.

Three nested models with four, five and six clinical predictors were compared in order to identify the one with the best goodness-of-fit. Model 1 was chosen because it presented the highest R^2^ value and was different from model 2 as indicated by the LR test. The selected model for case definition for ZIKV included six signs and symptoms and was converted in a score based on their coefficients (Fig 1). Considering the best cut-off value, a score equal or higher than 7·5 was predictive of Zika virus disease (Table 3).

**Table 3:** Clinical definitions of a suspected Zika case for surveillance purposes.

Case Definition	Description
**PAHO-2015**	Rash or elevated body temperature (>37·2^°^C) with at least one of the following symptoms: arthalgia or myalgia; non-purulent conjuntivitis or conjunctival hyperemia; headache or malaise
**CDC-2016**	One or more of the following: acute onset of fever (measured or reported), maculopapular rash, arthralgia, conjunctivitis, complication of pregnancy (fetal loss or microcephaly and/or intracranial calcifications), *Guillain-Barré* syndrome not known to be associated with another diagnosed etiology.
**PAHO-2016**	Rash (usually pruritic and macopapular) with two or more of the following signs or symptoms: fever (usually <38·5oC); conjuntivitis (non-purulent/hyperemic); arthalgia; myalgia; peri-articular edema
**ECDC-2016**	Rash with or without fever and at least one of the following symptoms: arthalgia or myalgia or non-purulent conjuntivitis/hyperaemia
**WHO-2016**	Rash and/or fever with at least one of the following symptoms: arthalgia or arthritis or conjuntivitis(non-purulent/hyperaemic)
**BRASIL(MoH)-2016**	Maculopapular rash pruritic with at least two of the following symptoms: fever or non-purulent conjuntivitis or polyarthralgia or periarticular edema
**Derived Score Prediction Model**	Score = 7*(maculopapularrash)+6*Temperature<37·5°C+4·5*itching+2·5*noanorexia+2*no petechiae+1*conjunctival hyperemia) RULE: Zika = Greater than or equal to the score 7·5

The multiple logistic regression results are presented in Table 4.

**Table 4:** Derived clinical prediction models of ZIKV infection.

Description of prediction model	OR		LR chi^2^/ pvalue	R^2^
		95%CI		
**Model 1—**Maculopapular rash + Itching + Fever +Conjunctival hyperemia + Petechiae + Anorexia			250·64/ 0·0000	0·417
Maculopapular rash	11·76	6·08–22·72		
Itching	7·79	4·35–13·95		
Conjunctival hyperemia	1·67	1·00–2·92		
Fever	0·10	0·04–0·22		
Petechiae	0·29	0·11–0·73		
Anorexia	0·23	0·13–0·41		
**Model 2—**Maculopapular rash + Itching + Fever + Petechiae + Anorexia			249·43/ 0·0000	0·413
Maculopapular rash	12·41	6·40–24·04		
Itching	8·35	4·67–14·90		
Fever	0·10	0·04–0·22		
Petechiae	0·31	0·12–0·79		
Anorexia	0·24	0·14–0·43		
**Model 3—**Maculopapular rash + Itching + Fever + Petechiae			224·98/ 0·0000	0·372
Itching	12·83	6·76–24·35		
Maculopapular rash	7·16	4·15–12·38		
Fever	0·10	0·05–0·21		
Petechiae	0·27	0·11–0·68		
**Likelihood-ratio test** (Model 1 *vs*. Model 2)			3·77/ 0·050	
**Likelihood-ratio test** (Model 1 vs. Model 3 vs· Model 2)			-382·48/ 1·0000	

As researchers consider the auROC measure of accuracy as the best measure of performance for diagnostic tests, we compared the model derived from our study with the other definitions used for a suspected case of Zika. The graph (Fig 1) indicates the superiority of the screening tool derived in our study in relation to others. It is noteworthy that the definition currently used in Brazil has an area of a little more than 0·5, which represents a lower power to discriminate suspected cases.

The suspected Zika case definitions were evaluated by significant differences in accuracy measurements (Table 5).

**Table 5:** Accuracy of Zika case definitions in simultaneous epidemics of dengue and chikungunya.

Case definition	Sensitivity		Specificity		DOR		auROC		R^2^	Brier score
	%	(95% CI)	%	(95% CI)		(95% CI)		(95% CI)		
**PAHO-2015**	81·3	54·4–96·0	10·9	4·5–21·2	0·53	0·12–2·14	0·461	0·355–0·567	0·008	0·750
**CDC 2016**	100·0	80·5–100·00	1·4	0·1–7·9	*	0·01–*	0·507	0·493–0·522	0·000	0·788
**PAHO-2016**	58·3	27·7–84·8	51·9	37·6–66·0	1·51	0·44–5·13	0·551	00·390–0·712	0·006	0·468
**ECDC-2016**	80·9	73·3–87·1	58·0	53·4–62·5	5·83	3·67–9·26	0·694	0·654–0·734	0·104	0·369
**WHO-2016**	75·6	67·4–82·5	63·5	58·9–67·8	5·37	3·48–8·28	0·695	0·652–0·737	0·102	0·338
**BRASIL(MoH)-2016**	28·6	20·9–37·3	97·3	95·3–98·6	14·40	7·26–28·40	0·629	0·589–0·669	0·113	0·179
Derived Score **Prediction Model**a	**86·6**	79·4–92·0	**78·3**	74·2–82·1	**23·40**	13·40–40·70	**0·903**	0·874–0·933	**0·417**	**0·096**

## Discussion

In this study, we formulated and tested a clinical based score to discriminate Zika cases from other febrile or exhantematic illnesses in a scenario where dengue and chikungunya viruses also circulate. The final score presented a sensitivity of 86·6% and a specificity of 78·3% and performed better than all other existing zika suspected case definitions to which it was compared.

In the definition proposed following our analysis, the symptoms most strongly associated with zika vírus disease were selected. These included presence of rash, pruritus, conjunctival hyperemia and absence of fever (axillary temperature less than 37·5°C), no petechiae, no anorexia.

Axillary temperature less than 37·5°C, petechiae and anorexia, signs and symptoms that are not included in existing suspected ZIKA case definitions, are included in the derived score prediction model with the best performance. This definition had the highest sensitivity and specificity, and also the highest diagnostic OR (23·4) value and auROC (0·903), as well as the lowest Brier score of 0·096.The Brier score approaching zero indicates that the Zika case infection definition proposed in our model is the one that best discriminates true cases of Zika symptomatic infection in our population.

The concurrent circulation of several arboviruses in many countries with endemic disease makes it difficult to differentiate between dengue, zika and chikungunya, which requires the use and standardization of more sensitive and specific criteria for definitions of suspected cases, in order to avoid notification errors and distortions in the analysis of arbovirus behavior.

The criterion derived from our prediction model did not include arthralgia, a symptom that was unable to discriminate between Zika and OFI, being frequent in both groups, with a prevalence of 69·6% and 56·3%, respectively. In fact although arthralgia is characteristic of Chikungunya, mainly due to its intensity and its longer duration (parameters not evaluated in the present analysis), it has been described with high frequency in other arboviral infections, as well as influenza and OFI [18]. In dengue, arthralgia is a symptom included in the WHO and Brazil MOH case definitions [19, 20] with frequencies above 70% being described in some studies [18, 21].

Fever (axillary temperature ≥37·5°C), a criterion present in most definitions, was inversely associated with Zika, as well as the presence of anorexia and petechiae, the more frequent manifestations of dengue fever and OFI prevalent in the study population.

Rash is not a mandatory symptom in the PAHO and CDC Zika case definitions, although it is listed, along with elevated fever, arthralgia and conjunctivitis as a common clinical manifestation of Zika virus infection. In both definitions, sensitivity was high (> 80%) and specificity was very low (<11%).

The problem of a criterion with high sensitivity and low specificity in countries with a triple arboviral epidemic is that it will not be able to discern which infection is responsible and will inevitably lead to a higher notification of Zika false positives, distorting the actual epidemiological data. This can lead to potentially unnecessary investigation of specific high risk groups, such as pregnant women for example, who will often turn up negative. In the CDC-2016 case definition, the simple presence of fever (measured or referred) defines a suspected case of Zika with a specificity of 1·4%, as fever is a very common symptom in almost all cases of Dengue and Chikungunya [19, 21] and common in most OFI in endemic and epidemic countries [18]. In the PAHO-2015 case definition which uses a mixed model, there was a slight increase in specificity (10·9%) by including, in addition to the presence of fever or rash, another symptom. However headache and malaise are considered non-specific and are also frequently seen in OFI, making it difficult to discern actual cases of Zika.

The two case definitions with the lowest sensitivity for detecting Zika cases were the ones used by PAHO and the Brazil–MoH. These fail the model for safe surveillance, as they may lead to a lower suspicion of true Zika cases which enables the detection of early cases and triggers an epidemic alert. However, although not sensitive, these definitions will likely report the highest number of true cases in triple-epidemic regions, because of acceptable degrees of specificity.

Despite the simultaneous circulation of more than one arbovirus [3, 22, 23] epidemics tend to produce different incidence patterns each year, with one arbovirus predominating over the others. The low sensitivity of Zika case definitions in Brazil allows many cases of Zika to be erroneously attributed to dengue fever. When using clinical criteria for the definition of a suspected case of dengue, 48% of confirmed cases of Zika in this study would have been reported as Dengue to the surveillance system. The misreporting of arboviral cases has serious consequences not only for the surveillance system but also for patient management and understanding of the pathogenesis of disease.

Our study, carried out in non-pregnant women and men, proposes three nested models. Maculopapular rash, itching, petechiae and temperature lower than 37·5C are signs common to all models. Anorexia was included in the first two models and non-purulent conjunctivitis, figures only in the first model. Model 1 showed a greater capacity of predicting Zika disease. Although this model requires a higher number of signs to be investigated in the potential Zika patient, it does not pose significant difficulty for its application in epidemiological surveillance.

The proposed definition originated from prediction models generated from data collected in our Acute Febrile Illness outpatient clinic over three years. These data are extremely rich because they reflect the epidemiological complexity of Rio de Janeiro in this period, which makes the differential diagnosis of viral diseases more difficult. On the other hand, it is not possible to determine if the predictive validity of the definition generated from these data will be reproducible in other scenarios, especially where there is circulation of other diseases that share some of Zika's symptomatology.

Surveillance systems use clinical and epidemiological criteria; therefore, in addition to clinical information, the epidemiological data on seasonality, laboratory confirmation of the first suspected cases, contact history, origin and dispersion rates are part of a suspected case definition. Some cases can be incorrectly classified, but this may have little impact in terms of public health, unless the classification system has so many limitations that high case numbers following epidemics with high attack rates are not perceived. The main objective in defining suspected Zika cases during outbreaks is to differentiate this condition from other epidemic arboviruses in a triple-epidemic scenario. Furthermore, the need to correctly identify circulating Zika virus is extremely important given its high pathogenicity to the developing fetus. As an unknown proportion of Zika cases are in fact asymptomatic, efforts to identify symptomatic disease are important and assist with sentinel surveillance.

## Conclusions

The analysis of clinical findings in a sample of laboratory confirmed cases of arboviral infections enabled us to generate a clinical case definition model for suspected Zika infections which has the best sensitivity and specificity. This model is useful for countries experiencing triple arboviral epidemics. Based on our findings, we suggest that the official definitions of suspected Zika cases be reviewed.
